# Protein engineering in the deep learning era

**DOI:** 10.1002/mlf2.12157

**Published:** 2024-12-26

**Authors:** Bingxin Zhou, Yang Tan, Yutong Hu, Lirong Zheng, Bozitao Zhong, Liang Hong

**Affiliations:** ^1^ Institute of Natural Sciences Shanghai Jiao Tong University Shanghai China; ^2^ Shanghai National Center for Applied Mathematics (SJTU center) Shanghai Jiao Tong University Shanghai China; ^3^ School of Information Science and Engineering East China University of Science and Technology Shanghai China; ^4^ Shanghai Artificial Intelligence Laboratory Shanghai China; ^5^ School of Electronic Information and Electrical Engineering Shanghai Jiao Tong University Shanghai China; ^6^ Department of Cell and Developmental Biology University of Michigan Medical School Ann Arbor Michigan USA; ^7^ School of Physics and Astronomy Shanghai Jiao Tong University Shanghai China; ^8^ Zhangjiang Institute for Advanced Study Shanghai Jiao Tong University Shanghai China

**Keywords:** artificial intelligence, geometric deep learning, protein engineering, protein language model, synthetic biology

## Abstract

Advances in deep learning have significantly aided protein engineering in addressing challenges in industrial production, healthcare, and environmental sustainability. This review frames frequently researched problems in protein understanding and engineering from the perspective of deep learning. It provides a thorough discussion of representation methods for protein sequences and structures, along with general encoding pipelines that support both pre‐training and supervised learning tasks. We summarize state‐of‐the‐art protein language models, geometric deep learning techniques, and the combination of distinct approaches to learning from multi‐modal biological data. Additionally, we outline common downstream tasks and relevant benchmark datasets for training and evaluating deep learning models, focusing on satisfying the particular needs of protein engineering applications, such as identifying mutation sites and predicting properties for candidates' virtual screening. This review offers biologists the latest tools for assisting their engineering projects while providing a clear and comprehensive guide for computer scientists to develop more powerful solutions by standardizing problem formulation and consolidating data resources. Future research can foresee a deeper integration of the communities of biology and computer science, unleashing the full potential of deep learning in protein engineering and driving new scientific breakthroughs.

## INTRODUCTION

Protein engineering, at the forefront of modern biotechnology, plays a pivotal role in addressing global challenges ranging from healthcare to environmental sustainability. Proteins exhibit versatile functionalities, serving as gene regulators, catalysts, and structural scaffolds in biological systems. Designing and enhancing proteins with desired functions holds immense potential for medical and industrial applications, such as drug discovery, design, and organ preservation. It also offers effective and innovative solutions to address emerging threats in sustainable development, such as carbon‐negative industries and plastic waste management, underscoring its significance in shaping the future of clean energy and human health.

The function of proteins is determined by their sequences and structures. Conventional computational and experimental protein engineering methods involve rational design, random mutagenesis, and saturation mutagenesis to introduce point mutations into wild‐type proteins, followed by extensive screening experiments to identify mutants that meet application requirements. Along with the rapid expansion of protein sequence and structure databases^1^, ^2^, ^3^, as well as the explosive development of computer science technologies, the latest research and development efforts have begun to integrate deep learning methods for protein function enhancement^4^, ^5^, ^6^, ^7^. These methods provide comprehensive guidance for protein engineering by learning the relationship between protein function and sequence/structure from extensive wild‐type proteins that exist in nature or from biological experiments.

The two fundamental forms of describing proteins, that is, sequence and structure, naturally give rise to two categories of deep learning models for protein representation encoding. Models for amino acid sequences typically employ protein language models, similar to analyzing tokens in natural language processing (NLP). The latest methods utilize Transformer^8^ to learn the pairwise relationships of amino acids and summarize the comprehensive role of each amino acid within the entire sequence. Two common sequence modeling strategies are masked language models^9^ and autoregressive sequence generation methods^10^. Alternatively, structure‐based deep learning models utilize geometric deep learning algorithms, such as convolutional neural networks (CNNs) and graph neural networks (GNNs), to analyze the backbone structure or surface of proteins. These methods typically require less data and lower model complexity, yet they have been developed slowly, until the emergence of structure prediction methods such as AlphaFold2^11^ and ESMFold^12^, due to the experimental difficulty of obtaining massive protein structure data compared to sequence information.

Due to the issue of data scarcity for experimental labels, the conventional protocol for training a deep protein representation learning model involves summarizing sequence and structure information using a pre‐training scheme. Such a scheme does not rely on labeled protein data from experiments, thus providing practical solutions for various downstream tasks, such as mutation scoring, function annotation, and property prediction. The associated zero‐shot predictions have seen success in protein engineering^4^, ^13^, ^14^ and protein design^5^, ^15^. Additionally, when a limited amount of experimental data is available, pre‐trained models can be continuously optimized to further enhance predictive performance^16^.

Developing artificial intelligence methods to solve synthetic biology problems has become an increasingly important research field. In this context, we review the main scientific questions and propose solutions in protein engineering, as well as discuss the remaining research problems to be explored. We start by formulating inference tasks related to protein sequences, structures, and functions, and we define common data processing methods based on accessible protein data modalities and available deep learning approaches. Subsequently, we enumerate the common deep learning pre‐training frameworks for different input types (e.g., amino acid sequence or structure), which fully utilize unlabeled data to extract hidden protein representations for downstream task predictions. Relevant prediction tasks and the datasets used for training and evaluation are listed in the section of “Benchmarks on the Downstream Tasks”. These three sections collectively frame the core steps for addressing various challenges in protein engineering using deep learning. At last, we outline future challenges and directions for the field's development.

## KNOWLEDGE ALIGNMENT: PROTEIN ENGINEERING AND DEEP LEARNING

In the context of deep learning, numerical representations of proteins are required. The different choices of input data modalities reflect the preferred inductive bias, which is closely related to the selection of deep learning techniques. As deep learning‐assisted protein engineering can be a highly interdisciplinary research area, both communities need to align the fundamental concepts before developing solutions to scientific problems. To this end, we start by framing the typical representations of proteins following the inference tasks associated with protein engineering. Subsequently, we summarize the conventional choices for protein characterization.

### The central dogma of protein engineering

**Figure 1:**
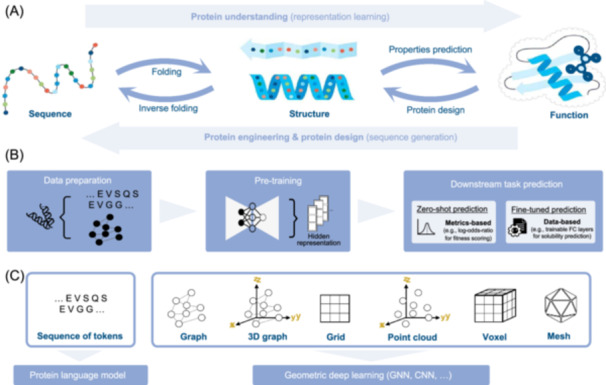
Fundamental concepts used in deep learning‐based protein understanding and engineering tasks. (A) The central dogma of protein engineering describes the relationship between protein sequence, structure, and function, the three key elements defining a protein. Prediction tasks have been formulated among these three modalities. (B) There are three steps in deep learning‐assisted protein engineering tasks, including numerical characterization of proteins for trainable data preparation, model pre‐training for hidden representation extraction, and downstream task prediction for specific applications. (C) There are different types of input data and selection of deep learning frameworks, which are determined by the inductive bias of the learning task. When amino acid sequences are considered sufficient for predicting the output label, sequences of tokens are sent to protein language models. Alternatively, when structures are accessible and important to the inference target, they are processed into geometric deep‐learning models, such as equivariant GNNs and 3D CNNs.

Proteins perform diverse functions in organisms, such as transport, storage, membrane composition, and enzymatic action. The function of a protein is determined by its structure, which is folded according to a specific sequence of amino acids (Figure 1A). The central dogma of proteins underlies the pathway of protein engineering and novel protein design^17^. We term the inference of structure and function from a given protein sequence as protein understanding. Conversely, determining the structure and sequence of a desired function (or structure) is referred to as protein engineering or protein design. In the context of protein engineering, a template protein and a function or property of interest are known, such as catalytic activity, thermostability, or solubility. The objective of engineering is to enhance the protein's performance by making local adjustments by substituting a few amino acids in the protein. Different from traditional methods like rational design and saturation mutagenesis, deep learning‐assisted engineering methods apply the principles of the protein engineering central dogma. By incorporating extensive wild‐type protein sequences and structures, the complicated projection of sequence‐structure‐function is inferred. This scheme guides the modification and design of proteins, achieving higher success rates and better assay performance at lower costs and faster speeds.

Unlike the massive amount of sequence and structure data, only a small fraction of proteins has been labeled with function and property annotations. Consequently, large‐scale powerful deep learning models are less popular in function prediction tasks in comparison to classic machine learning methods, such as gradient boosting and support vector machines. Additionally, many function annotations lack experimental validation, which hinders prediction models from achieving better accuracy due to their suboptimal inference power. Moreover, it is challenging to generalize quantified experimental metrics to other proteins or align them with different experimental environments, often leading models trained with supervised learning schemes to lack generalizability and practical significance. These problems have led to the development of self‐supervised learning schemes in recent years, in which a pre‐trained deep learning model^12^, ^18^, ^19^, ^20^, ^21^ only needs to learn from self‐labeled input data to obtain suitable hidden representations for subsequent downstream task predictions or fine‐tuning.

To translate between protein sequence, structure, and function, a standard deep learning solution for protein engineering consists of three steps, as illustrated in Figure 1B. First, the protein data are characterized into a numeric representation that can be processed by a model, such as a sequence of tokens, graphs, or voxels. Second, an appropriate encoding method (such as a protein language model or geometric deep learning) is selected based on the input data to transform the complex input data into vector representations. Since the learned vectors reside in a high‐dimensional space with no explicit physical meaning, they are referred to as hidden representations. Notably, training large models to learn expressive vector representations typically employs a pre‐training scheme since most protein data lack sufficient functional labels. Third, for a specified template protein to be engineered, a pre‐trained model is used to extract its hidden representation and score it. Depending on the accessibility of labels, as well as the practical requirements, when no labeled data are available, zero‐shot prediction is employed using measurements, such as entropy score and log‐odds ratio. When a small amount of labeled data is accessible, a feasible approach is to fine‐tune the model or scoring function to obtain more customized scores on a particular prediction task or assay.

### Numerical characterization of proteins

Before training deep learning models for protein understanding, the numerical representation of proteins has to be characterized first, such as the sequence and structural composition of proteins, as well as the explicitly defined molecular properties. The different ways of describing a protein reflect the preferred inductive bias and determine the choices of deep learning techniques in the later stage (Figure 1C). For instance, protein sequences are typically processed with language models to extract contextual representations along one‐dimensional chains, allowing long‐range relationships between different atoms or amino acids along the same chain to be captured. Alternatively, grid‐like representations are handled by convolutional networks, where atoms or amino acids within smaller receptive fields are more influential to the central instance. This section provides a summary of the common choices for characterizing proteins.

#### Sequence

The primary structure of a protein can be encoded as a unique sentence of amino acids. Excluding those that are uncommon or nontraditional, amino acids are classified into 20 different groups, and each type of amino acid is commonly designated by a unique one‐letter code, such as R for arginine and K for lysine. There are also statistical methods that merge common combinations of amino acids into phrases of tokens, which could represent meaningful units of proteins, such as secondary structures, motifs, or domains. Such enhanced representation improves the expressivity of amino acid sequences^15^, ^22^. In this way, amino acids, motifs, and domains in proteins are comparable to words, phrases, and sentences in human languages, in which sequential representations are typically encoded using language models, such as 1D CNNs^23^, ^24^ and attention mechanisms^18^, ^19^, ^25^. The former constructs the receptive field to efficiently compute the local environment of long sequences, and the latter analyzes the comprehensive relationship of the whole sequence at the cost of higher computational costs.

#### Structure

The sequential representation of molecules provides a detailed breakdown of their components. Alternatively, topological representations by graphs depict the intramolecular spatial interactions. In essence, a graph is composed of a set of nodes that represent the characters in the 1D sequence (atoms or residues), with edges connecting nodes based on identified relationships. These relationships can be either explicitly defined by biological connections (e.g., chemical bonds)^26^, ^27^ or implicitly defined by geometric connections (e.g., pairwise Euclidean distances in 3D space)^4^, ^28^. While graphs are arguably the most common choice for protein structures, there are also other variants of 3D geometric representation. If the pairwise connections are neglected, the group of entities is called a point cloud^29^, ^30^; if the nodes are assumed to align to the grid, it can be viewed as a voxel^31^; and if a molecule's shape is a major concern over its internal structure, a 3D mesh can be leveraged to describe the surface^32^. The choice of representation depends on the specific properties of interest, and all of them require consideration of rotation and translation equivariance in 3D space.

#### Molecular properties

Depending on the input unit and the potential application of the model, there are additional physicochemical or geometric features related to the functionality of the protein. Explicitly defining these features with domain knowledge could assist the model in learning the patterns and restrictions of protein construction. For instance, different types of amino acids correspond to the hydrophobicity, hydrophilicity, polarizability, and net charge of molecules, which can be important constraints for protein folding and functioning^33^, ^34^. At the structure level, the shape or conformation can usually be reflected by the 3D coordinates. Additional structure‐level summations include contacts, spatial distance, torsion angles, side‐chain direction, and pseudo force.

## PROTEIN REPRESENTATION LEARNING

A deep learning model learns vector representations for proteins in a hidden space, which is mapped by multi‐layer neural networks from the input data. The quality of these hidden representations determines the prediction performance for downstream tasks, such as function annotation. The choice of neural networks depends on the inductive bias of the prediction task, which is also reflected in the format of the input, as introduced in the previous section. For instance, amino acid sequences typically use Transformer‐based language models for encoding, while structure‐aware graph or voxel representations typically use neural message passing or 3D CNNs.

### Protein sequence encoding with language models

Similar to natural language, protein sequences can be represented as tokens. This consistency makes it a natural choice to adopt language models to analyze the token‐wise dependencies in protein sequences. The advances in gene sequencing technology bring an increasing number of high‐quality protein sequences, thereby expanding the corpus of protein sequences and enhancing the representation capabilities of large protein language models. Protein language models fit complex, large‐scale models using massive amounts of data to analyze the pairwise relationships of tokens (e.g., amino acids) in a sequence. Since they do not rely on prior knowledge, they have the potential to uncover more expressive representations beyond predefined topological relationships (for geometric deep learning models) or physicochemical descriptors (for classical machine learning models). However, this ability to extract comprehensive representations requires substantial resources, making it impractical in cases in which data are scarce or computational power is limited.

#### Encoder‐only models

**Figure 2:**
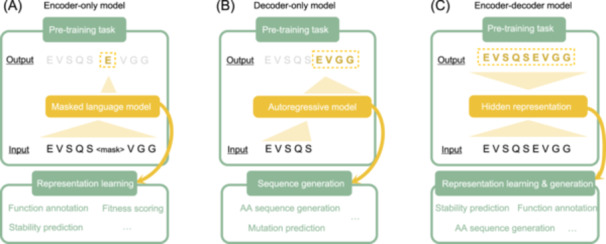
Three types of protein language models that extract vector representations from amino acid sequences. (A) Encoder‐only models take the MLM framework, whose pre‐training task is to recover the randomly masked tokens (amino acids). The obtained residue‐level vector representation can be used for downstream label prediction tasks. (B) Decoder‐only models iteratively recover the i+1 th amino acid based on the first i amino acids. The pre‐trained models usually perform generative tasks. (C) Encoder‐decoder models find expressive embedding for amino acid sequences by training the model to reconstruct the extracted sequences. The embedding can be used for both representation learning and sequence generation.

The first mainstream category of pre‐training protein language models is encoder‐only language models (Figure 2A), which, in NLP tasks, are commonly used for text understanding and representation learning. An encoder model maps the input text sequence to a set of vectors. The projection rules are learned extensively from a large corpus through self‐supervised learning. The output of the last encoding layer provides the amino acid representation of the protein in a hidden space, which is expected to capture the implicit main characteristics of the protein and can be used for downstream tasks, such as mutation effect prediction and function prediction. The majority of encoder‐only protein models follow the Transformer‐based BERT framework^9^.

Two types of BERT‐style learning tasks in NLP are masked language modeling (MLM) and next sentence prediction. MLM is the preferred choice for existing protein language models because next sentence prediction is used to predict the relationship between two sequences of text, and annotating sufficient contextual relationships is nearly impossible for proteins. During the training phase, MLM randomly masks a subset of tokens (usually 15% of all the amino acids), and the training objective is to minimize the difference between the prediction of the masked amino acid and the ground truth, i.e.,

(1) $${ℒ}_{MLM}=E_{x\sim X}E_{M}\sum_{i\in M}-\;\log\;p(x_{i}|x_{/M}).$$

Here, $x_{/M}$ denotes the protein sequence excluding the masked tokens, where the sequence x∈X is extracted from the population dataset X. The conditional probability $p\left(x_{i}|x_{/M}\right)$ of the ith token $x_{i}$ is based on the unmasked amino acids $x_{/M}$. The training objective is to minimize the negative log‐likelihood of correctly identifying all the masked true amino acids based on the unmasked part. This requires the model to effectively learn the relationship between masked and unmasked tokens.

MLM (and its variants) has become the predominant choice for protein language representation model work. An iconic example is the ESM series. ESM‐1b^18^ analyzes the biochemical pattern and evolutionary homology of a particular protein and customizes hidden representation for it. ESM‐1v^19^ applies the MLM training framework to zero‐shot mutation effect prediction with log‐odds scoring functions. Multiple sequence alignment (MSA)‐Transfomer^35^ integrates evolutionary information from MSA sequences with column attention. ESM2^12^ constructs MLM tasks on Uniref50 with Roformer rotational position encoding^36^. The learned representations are now widely used for MSA‐free folding (ESMFold) and downstream task prediction. Similarly, ProtBert and AIProtBert from the ProtTrans^25^ series employ modified BERT architectures with relative positional encoding. PETA^22^ constructs Roformer‐based architecture and investigates the impact of different segmentation methods and vocabulary sizes.

In addition to BERT and the BERT variants, other methods introduce additional neural network layers to enhance self‐attention propagation. For instance, ProteinBERT^24^ replaces self‐attention operations with wide and narrow 1D CNN layers and enhances representation learning by combining language modeling and gene ontology (GO) annotation prediction tasks. CARP^37^ trains a CNN‐based MLM, which achieves comparable performance to self‐attention with higher computational efficiency, accommodating longer protein sequences. Alternatively, embedding ability is enhanced with structural sequences or structure‐aware tasks. SaProt^38^ trains a structure‐aware version of ESM2 with the vector vocabulary of amino acid composition from Foldseek^39^. ProSST^40^ further employs disentangled attention to the amino acid sequence and vectorized local structure representation to improve prediction performance in zero‐shot mutation prediction tasks.

#### Decoder‐only models

Decoder‐only models in NLP are widely used for generative tasks, such as text generation, expert dialogue, and code generation. The objective is to generate reasonable text sequences autoregressively with optional prompts. In protein sequence generation, a model is trained to design novel sequences with particular functionalities. Currently, mainstream protein language models adopt GPT‐style^10^ architecture with the Transformer decoder, which is trained to optimize the next‐token prediction, that is,

(2) $${ℒ}_{GPT}=-\sum_{i=1}^{n-1}\log\;p(s_{i+1}|s_{1:i};\theta ),$$

where $s_{i}$ denotes the ith amino acid in the sequence, θ is learnable parameters, and $p\left(s_{i+1}|s_{1:i};\theta \right)$ represents the probability of the next amino acid $s_{i+1}$ based on all the previous i amino acids $s_{1:i}$.

ProtGPT2^41^ is trained on the wild‐type protein sequence space to guide the model to autoregessively generate novel amino acid sequences that resemble natural proteins. ProGen^15^ enhances the generation reliability through context learning and model fine‐tuning on sequences from close functional families. RITA^42^ proposes a series of autoregressive models of varying scales to investigate the relationship between model size and the quality of generated protein sequences. Tranception^23^ and TranceptEVE^43^ utilize the GPT2 architecture to learn k‐mer protein tokens and search MSA sequences to enhance the learning of specific protein functional features. PoET^44^ clusters wild‐type protein sequences and designs autoregressive models based on protein family pairs to enhance the language model's generation capability for specific functions.

Nevertheless, the validation for protein sequences is much more challenging than natural language due to the complexity and high cost of biological experiments. Alternatively, in‐silico evaluation from structural and functional perspectives is commonly adopted to assess the reliability of generated sequences. With fast and accurate predictions of foldable structures, such as AlphaFold2 or ESMFold, the generated results can be assessed qualitatively based on whether the 3D structure is globular^41^ or quantitatively on the foldability based on predicted pLDDT scores^15^. At the function level, downstream tasks such as fitness scoring and function annotation are commonly incorporated for evaluation^23^, ^44^.

#### Encoder‐decoder models

Transformer‐based encoder‐decoder models are designed for end‐to‐end machine translation tasks in NLP. The joint training model construction can be used for both representation learning and text generation. A handful of protein language models construct encoder‐decoder architectures or their variants, aiming to achieve better sequence representation or generation capabilities. The general encoder‐decoder architecture of a Transformer model, at the ith layer, is defined as

(3) $$H_{i}=\mathrm{Encoder}(s_{i:n};\;\theta_{\mathrm{encode}})$$

(4) $$p{(s}_{j}|s_{1:j-1},H)=\text{Decoder}{(s}_{1:j-1},H;\;\theta_{\text{decode}}).$$

In essence, the encoder maps an input sequence $s_{1:n}$ into the hidden state $H_{i}$, and the decoder predicts the next amino acid $s_{j}$ based on the sequence context $s_{1:j-1}$ and the hidden state of the sequence H. The training target is to learn $\theta_{\mathrm{encode}}$ and $\theta_{\mathrm{decode}}$ that lead to the most similarly decoded sequences with respect to the input sequences. xTrimoPGLM^45^ utilizes perturbation pre‐training and multimodal learning from the GLM^46^ to build a model framework with over 100 billion parameters for protein sequence design and downstream prediction tasks. Ankh^47^ improves the performance of the MLM‐based pre‐training model with an asymmetric encoder–decoder architecture. ProstT5^21^ extends ProtT5^25^ to a structure‐aware protein language model with Foldseek embedding^39^. Additionally, MSA is another type of commonly employed information when enhancing the model performance in protein engineering or protein design. For instance, MSA2Prot^48^ constructs MSA‐to‐protein conditional generation by learning from MSA encoding and Pfam^49^ labels.

### Protein structure encoding with geometric deep learning

**Figure 3:**
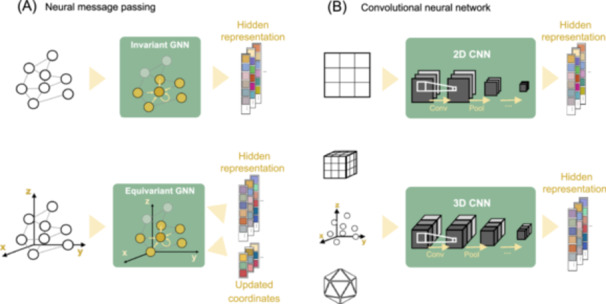
A depiction of structure‐based models for protein embedding. (A) Neural message passing for graph input. The (permutation) invariant GNN layers analyze the connection of entities, while the (rotation) equivariant GNN layers update node representation with their 3D coordinates in the Euclidean space. Both graph convolutional layers summarize residue‐level hidden representations. (B) Convolutional neural networks. When the input protein is presented in grids, 2D CNNs propagate pixel representations by convoluting the neighboring pixels of a certain receptive field, similar to 2D images. For 3D objects such as voxels, point clouds, or mesh, the convolutional layers generalize to a higher dimension (3D) with similar operations. The output hidden representation, from both GNNs and CNNs, can be provided at either the residue level or the protein level (with pooling layers), depending on the demand of downstream tasks.

The topology of proteins is crucial for understanding their functionality. To characterize protein data, various inductive biases can be incorporated, such as 3D density mappings with CNNs and interactive propagation with GNNs. The basic structural representation units of proteins are presented in different ways to fit the nature of the network propagation rules. For instance, GNNs learn from graphs, with amino acids frequently being the minimum units connected based on their Euclidean distance. Alternatively, CNNs learn proteins represented as grids, voxels, point clouds, or mesh (Figure 3). While both CNNs and GNNs are methods for handling structural data, CNNs are more commonly used for regularly arranged data, such as those fit in grids or meshes. However, the dense connections among entities make it difficult to capture long‐range spatial relationships, even though they can be essential for proteins. On the other hand, GNNs are a natural choice for loosely connected, non‐Euclidean relationships and can incorporate prior structural knowledge to reduce the dependencies for massive training datasets. However, GNNs have trouble communicating between unconnected nodes in a single layer, and the GNN community is smaller than the computer vision community, resulting in fewer mature and readily available models or tools compared to CNNs.

#### Neural message passing

Message passing^50^ is a widely adopted concept in geometric deep learning, which formulates stepwise propagation and aggregation of information from a node to its nearest neighbors. For a graph, the hidden representation $H_{i}^{\left(l+1\right)}$ for the ith node in the (l+1)th layer is updated by

(5) $$H_{i}^{\left(l+1\right)}=\gamma \left(H^{\left(l\right)},{⊕}_{V_{j}\in N\left(i\right)}\phi \left(H_{i}^{\left(l\right)},H_{j}^{\left(l\right)},A_{ji}\right)\right),$$

where the information from all one‐hop neighbors $V_{j}\in N(i)$ in $H^{\left(l\right)}$ is aggregated using a differentiable and permutation‐invariant function ⊕, such as summation, average, or maximization. The pairwise connections of nodes are defined by the input adjacency matrix A, where $A_{\mathrm{ji}}$ refers to the connection from node j to node i. The functions γ,ϕ represent another two differentiable transformations. The spatial position of amino acids can be represented by the dihedral angles on the main chain, that is, the directional relationship of the heavy atoms N, C, and O.

GNNs have become the predominant choice for structure‐informative tasks, such as protein inverse‐folding and protein‐ligand interaction. Among these models, graph convolution networks^51^ and graph attention networks^52^ are widely adopted. Additionally, many models make modifications to the message‐passing propagation rules for better performance. For instance, ProteinMPNN^53^ employs an encoder‐decoder GNN architecture to update the node and edge representations of the protein graph. GraphTrans^54^ designs multi‐head self‐attention for k‐nearest neighbor (kNN) graphs for autoregressive amino acid sequence generation. AlphaDesign^55^ further extends GraphTrans with a simplified graph attention encoder and a non‐autoregressive decoder consisting of two 1D CNNs. In addition to propagating among spatially adjacent nodes, GCA^56^ incorporates self‐attention layers on all nodes to aggregate global patterns of proteins. GearNet^57^ combines spatial and sequential information and designs message‐passing propagation rules for different types of edges in a relational graph.

As a protein's atomic dynamics remains unchanged regardless of its translation or rotation within a system^58^, several propagation rules incorporate the inductive bias of symmetry into structure‐based models, requiring the learned 3D protein transformations to belong to the Special Euclidean group SE(3)^59^, which is the set of rotation and translation equivariant transformations in 3D space, that is, (ℛ,t)X=ℛX+t with some rotation matrix ℛ∈SO(3) and translation vector $t\in R^{3}$. These algorithms avoid expensive data augmentation and typically need the propagation to consider positional encoding of 3D coordinates $X^{\prime }\in {\mathbb{R}}^{n\times 3}$ ^60^. There are various approaches to implementing equivariant geometric GNNs^61^, ^62^, ^63^. Particularly, EGNN has seen applications in generative models. CPDiffusion^5^, ^64^, ^65^ employs EGNN to integrate the backbone and secondary structure conditions during sequence generation. RFDiffusion^66^ constructs SE(3)‐Transformer with tensor field networks^67^ for structure denoising. Another alternative architecture is GVP, which incorporates both scalar and vector features at propagation and has shown advantages for structure prediction^68^, substructure alignment^28^, inverse folding^62^, ^69^, and downstream properties prediction tasks^70^, ^71^.

#### CNNs

CNNs were initially developed for image processing, and they are known for adequately capturing the information flow along a local grid. This property makes CNNs a suitable choice for certain structure‐sensitive tasks, such as binding prediction. Among various models for handling pixelated proteins, the most common choice is 3D CNN due to its translation invariance. The convolutional operation of a 3D CNN layer on voxels is

(6) $$O(i,j,k)=\sum_{m=0}^{M-1}\sum_{n=0}^{N-1}\sum_{p=0}^{P-1}M(i+m,j+n,k+p)K(m,n,p).$$

Here, O(i,j,k) represents the element at position (i,j,k) in the output feature map, M is the input feature map, K is the 3D convolution kernel, and M, N, and P are the depth, height, and width of the kernel.

Through multi‐layer convolutions on voxels of 3D protein images^72^, ^73^, ^74^, distance maps^75^, ^76^, or surfaces^77^, local patterns at different scales are detected, such as atom‐level, sequence‐level, and secondary‐structure‐level constructions. For instance, ProDCoNN^72^ trains nine‐layer 3D CNNs for inverse‐folding and mutation prediction. Alternatively, DenseCPD^73^ conducts similar tasks with DenseNet to achieve a more expressive representation. In addition to sequence generation, other studies leverage 3D CNNs to extract the local chemical or physical properties and predict the rotamer angles of a backbone^74^, ^78^ or categorize their roles in a metabolic pathway^79^. The convolutional layers have also been used to extract protein surface structures. MaSIF^80^ describes the surface of protein by mesh and extracts descriptors from geometric and chemical node features within multiple overlapping patches of the same radius for downstream tasks. To improve computational efficiency, dMaSIF^81^ further represents the protein molecular surface as oriented point clouds and uses quasi‐geodesic convolutions to extract molecular surface features.

### Protein multimodal and multiscale joint encoding

There is potential for integrating protein descriptions into various modalities and scales to uncover the mechanisms behind protein functionalities^34^, ^82^. A handful of previous studies feature different morphologies of proteins, of which sequence and structure representations are arguably the two most popular options. Large, pre‐trained language models such as ProtBERT^25^ and ESM‐2^83^ are used for sequential representations, while GNNs and CNNs can encode local environment information for structure representations. ProREM^84^ leverages protein language models to encode sequences, local structures, and evolutionary information to greatly improve the performance of mutation predictions. ProLLaMA^85^ modifies LORA^86^ to adapt its training framework for tasks such as protein property prediction and protein design. In downstream tasks, various algorithms such as multilayer perceptrons (MLPs)^87^, ^88^, autoencoders^30^, ^89^, or other specially designed methods^90^, ^91^, integrate separate embeddings from different modalities into a single‐vector representation. MSA can also enhance sequential inputs to identify conserved and variable regions of proteins from homologous sequences^11^, ^19^, ^35^. Other research seeks versatile modalities. HOLOPROT^32^ represents a protein's surface and structure; the former generates the spherical relationship of residue nodes through a triangulation algorithm, and the latter captures four levels of a protein structure by a residue graph with multi‐scale message passing networks. {Transformer‐M}^92^ establishes atomic‐level descriptions for a protein by incorporating 1D (e.g., physiochemical properties), 2D (e.g., sequential distance), and 3D (e.g., Euclidean distance) channels.

In addition to different modalities, describing proteins from different scales and obtaining more detailed representations while minimizing costs can be highly advantageous. Several methods have been proposed to extract feature representations for proteins and ligands at different levels. For example, DeepFusion^93^ generates feature representations for drugs and proteins using precomputed global structural similarity matrices and local chemical substructure semantic features. To encode structure representations at multiple scales, a coarse‐grained GNN handles node features and edge connections at coarser levels with gated recurrent units^94^. AGL‐Score^95^ and MWCG^96^ construct multiscale weighted colored subgraphs with atomic types and extended connectivity interactive features to capture intramolecular interactions between proteins and ligands. MSSA‐Mixup^97^ captures internal dependencies between different scales of graph signals by graph wavelet theory using self‐attention and^98^ develops the persistent path‐spectral model that characterizes the data at multiple scales during the filtration process.

## BENCHMARKS ON THE DOWNSTREAM TASKS

Evaluating inference performance is a crucial step in ensuring that a deep learning model has been adequately trained. Given the high cost of wet lab experiments, experimentally validating every developed model is impractical. Instead, an alternative approach is to initially validate the expressivity of the hidden representations extracted by the models on downstream tasks with open benchmark datasets (Table 1). For pre‐trained deep learning methods, a common practice to ensure that the model outputs the desired labels is to fine‐tune the model architecture or parameters based on the initial construction to adapt to specific downstream tasks and datasets. This is particularly important when the output labels change. For instance, in self‐supervised learning, the outputs are generally residue‐level probabilities of AA types, whereas the downstream task may require a regression score for the entire protein to represent the property of interest. In such cases, additional propagators are often attached, such as geometric encoders^20^, ^57^, ^145^ or cross‐modality aggregators^33^, ^34^, ^82^.

**Table 1:** Benchmark datasets for protein learning tasks.

Dataset (version)	Size	Resource
General benchmark		
PETA^22^	15 tasks	https://github.com/ginnm/proteinpretraining
PEER^99^	14 tasks	https://torchprotein.ai/benchmark
ProteinGLUE^100^	7 tasks	https://github.com/ibivu/protein-glue
BioMap^45^	26 tasks	https://www.biomap.com/sota/
Mutation effect prediction		
ProteinGym v1^101^ (DMS substitutions)	217 assays	https://proteingym.org/
ProteinGym v1^101^ (DMS Indels)	66 assays	https://proteingym.org/
DeepSequence^102^	33 assays	https://github.com/debbiemarkslab/DeepSequence
Envision^103^	21,026 variants	https://envision.gs.washington.edu/shiny/envision_new/_
FLIP^104^	3 tasks	https://benchmark.protein.properties/
FS‐mutant^105^	11 assays	https://github.com/ginnm/fs-mutant
SoluProtMutDB^106^	32,992 variants	https://loschmidt.chemi.muni.cz/soluprotmutdb/
DTM, DDG^20^	102 assays	https://github.com/tyang816/ProtSSN
FireProtDB^107^	15,987 experiments	https://loschmidt.chemi.muni.cz/fireprotdb/
ProThermDB^108^	32,000 experiments	https://web.iitm.ac.in/bioinfo2/prothermdb/
Structural homology detection		
PDB^2^ (2023.01)	215,100 proteins	https://wwpdb.org/
CATH‐Plus^109^ (v4.3)	500,238 domains	https://www.cathdb.info/
AlphaFold DB^110^	200 M proteins	https://alphafold.ebi.ac.uk/
Malidup^111^	241 domain pairs	http://prodata.swmed.edu/malidup/
Malisam^112^	130 motif pairs	http://prodata.swmed.edu/malisam
SWISS‐MODEL^3^	2,751,760 models	https://swissmodel.expasy.org/repository
Pfam^49^ (v36.0)	20,795 families	http://pfam.xfam.org/
InterPro^113^ (2024)	45,000 items	https://www.ebi.ac.uk/interpro
ModBase^114^	1,262,629 models	https://modbase.compbio.ucsf.edu/
Protein solubility prediction		
TargetTrack^115^	350,000 proteins	https://zenodo.org/records/821654
PROSO 2^116^	82,000 proteins	http://mips.helmholtz-muenchen.de/prosoII
DeepSol^117^	69,420 proteins	https://machinelearning-protein.qcri.org/index.html#!pages/deepsol
DeepSoluE^118^	11,436 pairs	http://lab.malab.cn/∼wangchao/softs/DeepSoluE/
Protein‐Sol^119^	2395 pairs	https://protein-sol.manchester.ac.uk/
SoluProt^120^	11,436 pairs	https://loschmidt.chemi.muni.cz/soluprot/
eSOL^121^	1128 pairs	http://www.tanpaku.org/tp-esol/download.php?lang=en
Protein localization prediction		
DeepLoc‐1^122^	13,858 proteins	https://services.healthtech.dtu.dk/services/DeepLoc-1.0/
DeepLoc‐2^123^	28,303 proteins	https://services.healthtech.dtu.dk/services/DeepLoc-2.0/
LOCATE^124^	58,128 mouse proteins,	http://locate.imb.uq.edu.au/
WoLF PSORT^125^	64,637 human proteins	https://www.psort.org/
iLoc‐Euk^126^	17,217 proteins	http://icpr.jci.edu.cn/bioinfo/iLoc-Euk
Hum‐mPLoc 2.0^127^	7766 proteins	http://www.csbio.sjtu.edu.cn/bioinf/hum-multi-2/
Plant‐mPLoc^128^	3106 proteins	http://www.csbio.sjtu.edu.cn/bioinf/plant-multi/
Protein annotation prediction		
Gene Ontology^129^	‐	https://geneontology.org/
Enzyme Commission^130^	‐	https://enzyme.expasy.org/
PANTHER^131^	2,617,023 genes	https://pantherdb.org/
BioCyc^132^	20,050 pathways/genomes	https://biocyc.org/
Swiss‐Prot^133^	571,609 proteins	https://www.uniprot.org/
UniProt^134^	25,063,527 proteins	https://www.uniprot.org/
Compound‐protein interaction		
BindingDB^135^ (2024.5)	2,889,422 pairs (9314 proteins, 1,246,739 compounds)	https://www.bindingdb.org/rwd/bind/index.jsp
ChEMBL^136^ (2024.3)	20,772,701 pairs (15,598 proteins, 2,431,025 compounds)	https://www.ebi.ac.uk/chembl/
PDBbind‐CN^137^ (2020)	23,496 complexes, 326 kinases	http://www.pdbbind.org.cn/
KLIFS^138^ (2024.6)	4148 ligands, 6667 PDB structures	https://klifs.net/
DrugBank^139^ (2024)	>28,000 drug–protein interactions	https://www.drugbank.com/
BioLiP2^140^ (2024.5)	87,3925 ligand–protein interactions	https://zhanggroup.org/BioLiP/index.cgi
Protein‐protein interaction		
STRING^141^ (2023.7)	59,309,604 proteins, 27,541,372, 833 interactions	https://string-db.org/
BioGRID^142^ (2024.3)	2,780,952 protein/genetic interactions	https://thebiogrid.org/
IntAct^143^ (2024)	124,275 proteins, 1,572,071 binary interactions	https://www.ebi.ac.uk/intact
HPRD^144^ (2006.10)	9427 proteins, 36,617 human protein–protein interactions	http://www.hprd.org/

### General benchmark

A handful of integrated benchmark datasets have been proposed for computational protein engineering. They usually encompass diverse prediction tasks and can be implemented for testing both pre‐trained and fine‐tuned models. For instance, TAPE^146^ consists of three structure prediction and two fitness prediction datasets. PEER^99^ comprises 17 protein‐related tasks, such as protein function prediction, localization prediction, and solubility prediction. ProteinGLUE^100^ includes seven domain‐level downstream tasks for assessing self‐supervised protein representation learning. PETA^22^ contains 15 downstream tasks and 33 datasets associated with protein‐protein interaction and mutation prediction. BioMap Leaderboard^45^ introduces a comprehensive leaderboard of life science covering antibodies, enzymes, and other general proteins. It is specifically designed for assessing the fine‐tuning performance of pre‐trained protein language models.

### Mutation effect prediction

Developing deep learning models to provide reliable prediction of mutation effects is essential for protein engineering as it guides modifying proteins toward enhanced functionalities, such as activity, stability, and yield. The emergence of deep mutational scanning and other high‐throughput techniques has greatly accelerated the enrichment of protein mutation databases. The related benchmarks can generally be divided into two main categories: zero‐shot prediction and supervised prediction. They utilize different evaluation metrics, focus on different assays, and are primarily applied to different types of models. To date, more than two million assessed mutants from over 200 deep mutational scanning assays have been recorded and standardized for evaluation, spanning different taxa and functions^23^, ^102^, ^103^. In particular, ProteinGym v1^101^ is the most comprehensive benchmark dataset that includes both single‐site and multiple‐site mutations of substitutions and indels. There are also specialized benchmarks for single amino acid variation for solubility^33^, ^106^, thermostability^20^, ^107^, ^108^, and other properties^34^. FLIP^104^ evaluates supervised prediction performance on single‐site and double‐site classification tasks. FS‐mutant^105^ further categorizes epistatic and non‐epistatic sample datasets for few‐shot learning evaluation.

### Structural homology detection

Analyzing the energy and physicochemical properties of protein crystal structures provides a better understanding of protein mechanisms. Meanwhile, analyzing structural homology establishes more robust search methods to locate proteins from the same functional family with lower sequence identity, which is desired for applications such as protein design, evolutionary analysis, and enzyme function discovery. Existing benchmarks are usually protein structures with hand‐crafted or computational labels associated with structural or functional families. PDB^147^ is the largest global protein structure database that collects experimentally solved protein structures. Pfam^49^ provides a comprehensive collection of protein families and domains with hidden‐Markov‐model‐based MSA, and it now has been moved to InterPro^113^. Malidup^111^ contains 241 manually aligned homologous structural domains, which have different functions and highly divergent sequences. Malisam^112^ is a structural analog dataset consisting of 130 pairs of structurally similar motifs that are difficult to align using sequence‐based methods like BLAST and HMMER. CATH^109^ contains over 500,000 labeled protein domains from PDB, which has four categories based on class, architecture, topology, and homologous superfamily. SWISS‐MODEL^3^ pairs the predicted structure of protein sequences with the experimented protein structures of their homologies through sequence alignment. SCOPe^148^ analyzes the relationships and evolutionary paths of proteins through multi‐level protein structures and functions (such as families, superfamilies, and domains). ModBase^114^ provides predicted protein structures from sequence alignments, which are used to identify functional domains and predict the functions of unknown proteins.

### Function prediction

In addition to modifying proteins for specific functions or properties by substituting or inserting/deleting amino acid sequences, it is also possible to predict the corresponding properties or functions of an input protein sequence (and/or structure). This allows for computational screening or evaluation of newly designed or modified proteins, helping to preliminarily eliminate potentially problematic products and thus reduce the experimental failure rate.

#### Solubility prediction

Understanding solubility is essential for successful protein engineering as proteins are required to be expressible and soluble before functioning, and higher solubility potentially enhances industrial production yields. Deep learning models benefit from rich multi‐modal solubility datasets with computational and experimental sources. The cDNA^149^ sequence data collects solubility experimental data based on cDNA sequences, allowing the study of direct mappings from DNA sequences to protein solubility. TargetTrack^115^ thoroughly records each experiment's results and historical statuses, such as selected, soluble, crystalized, and in PDB. PROSO II^116^ integrates data from other databases such as PDB and TargetTrack, offering a comprehensive dataset with over 60,000 plausibly soluble and nonsoluble entries. DeepSol^117^ and DeepSoluE^118^ further guarantee heterogeneity and reduce class imbalance based on PROSO II with redundancy removal and filtration. eSOL^121^ collects thousands of solubility records from *Escherichia coli* proteins synthesized via the PURE system. Other work^119^, ^120^ builds web servers for solubility prediction and dataset download.

#### Localization prediction

Predicting the subcellular localization of proteins is a crucial aspect of cell biology and functional genomics. It is essential for understanding protein function, analyzing cellular signaling pathways, and gene expression regulation. LOCATE^124^ offers extensive data on the localization of protein isoforms in mouse and human tissues, including subcellular localization predictions, experimental image data with automated classification tags, and predictions of protein sorting signals. PSORT^77^ compiles a wealth of predictive and experimental labels for protein sequences, focused on their subcellular localization. The DeepLoc series^122^, ^123^ differentiates membrane proteins, including various datasets for binary classification, single‐label ten‐class classification, and multilabel multiclass classification. There are also databases tailored to the subcellular localization of specific species, such as fungi^126^ and plants^128^, and databases for animals and humans. These datasets and tools provide comprehensive information on protein localization, supporting research in protein function, cell biology, and the elucidation of disease mechanisms.

#### Annotation prediction

Accurately annotating the functions of genes and proteins helps researchers understand the mechanisms of biomolecules, metabolic pathways, and biological networks. Two of the most commonly used annotation systems are GO^129^ and EC (Enzyme Commission) numbers^130^. GO annotates protein functions based on biological processes, molecular functions, and cellular components, and the annotated proteins are from different species. EC numbers, in comparison, provide a numerical classification scheme for enzyme‐catalyzed chemical reactions. It assigns the same EC number to enzymes catalyzing the same reaction, no matter whether they originate from the same organism or not. Other well‐known open‐access functional annotation databases include PANTHER (Protein ANalysis THrough Evolutionary Relationships)^131^, which integrates gene ontology and evolutionary relationships of gene families to provide a functional classification of genes. BioCyc^132^ combines metabolic pathways and gene function annotations to support the analysis of genomes and metabolic networks. Additionally, large‐scale general databases such as Swiss‐Prot/UniProtKB^133^ and UniProt^134^ offer more comprehensive functional annotations. These databases integrate experimental data and literature information to provide detailed protein function annotations, serving as reliable reference data for functional annotation predictions.

### Protein binding

In addition to the properties of individual proteins (amino acid composition, 3D structure, and function), the interactions between proteins and other molecules (such as compounds, proteins, and antibodies) are also a crucial research area of protein engineering. They have significant implications for explaining disease mechanisms, discovering unknown disease genes, and designing more specific binders, among other applications.

#### Compound–protein interaction

Compound–protein interaction is one of the key research areas in biochemistry and drug development, providing critical support for improving metabolic regulation and screening new drugs. Several existing datasets offer structural and dynamic information on compound‐protein interaction and provide detailed annotations on binding affinity, activity, and other aspects. BindingDB^135^ contains interaction data for approximately 9300 protein targets and 1.2 million ligands sourced from public patents and the literature, totaling around 2.9 million binding affinity pairs. ChEMBL^136^ also integrates information from other public databases, including biological activity data for over two million compounds from around 15,000 targets. PDBbind^137^ extracts approximately 20,000 protein–ligand complexes from the PDB Bank and annotates corresponding binding constants such as Kd, Ki, and IC50, providing detailed binding affinity data. Additionally, there are several datasets focused on more specific research subjects. For example, KLIFS (Kinase‐Ligand Interaction Fingerprints and Structures)^138^ contains about 300 kinases, 4000 ligands, and 7000 PDB structures. DrugBank^139^ includes information on around 5000 protein targets, combining drug chemistry with a bioinformatics database. BioLiP^140^ offers detailed information on over 800,000 ligand‐protein complexes, including extensive annotations and cross‐references to other databases, such as ligands, catalytic sites, EC numbers, and GO terms.

#### Protein–protein interaction

Protein–protein interaction research encompasses binding score prediction or binary interface label assignment at the atom, residue, or protein levels. Existing studies frequently combine expert knowledge with computational induction and integrate protein‐protein interaction data from experiments, literature, computer simulations, and other databases. Differences between various databases mainly lie in the types of proteins they include and the scope of their annotation labels. STRING (Search Tool for the Retrieval of Interacting Genes/Proteins)^141^ consolidates a vast amount of in vivo protein data and annotates protein function labels derived from experiments, literature mining, and algorithmic predictions. BioGRID (Biological General Repository for Interaction Datasets)^142^ offers a comprehensive and continuously updated resource of protein‐protein interaction data, aggregating interaction data from various experimental methods, such as yeast two‐hybrid system, high‐throughput screening, and co‐immunoprecipitation. IntAct^143^ is a database actively maintained by the European Bioinformatics Institute, which provides abundant experimental details in addition to protein interaction labels. HPRD (Human Protein Reference Database)^144^ specifically addresses human protein interactions and includes annotations on protein functions, modification states, and subcellular localization.

## CONCLUDING REMARKS

Deep learning has revolutionized protein engineering, injecting fresh momentum into the exploration and manipulation of protein functions. By harnessing cutting‐edge techniques, such as protein language models and geometric deep learning, researchers have developed powerful algorithms for understanding the relationships between protein sequence, structure, and functions by learning from large‐scale wild‐type protein databases. The introduction of these tools has witnessed the synergy of emerging technologies driving the rapid advancement of biology, particularly in discovering better proteins and assisting virtual screening schemes at lower time and economic costs, even without extensive experimental data or rich domain knowledge. Moreover, these tools have further advanced researchers' understanding of proteins from mechanisms to functions, demonstrating deep learning's considerable promise for tackling complex biological problems.

Despite the significant development and notable success stories, the evolution of deep‐learning‐assisted protein engineering is still in its early stages, with many crucial challenges remaining to be addressed. One important issue is the explainability of large deep‐learning models. Explainable artificial intelligence (AI) is considered a crucial aspect of assessing the reliability of models, especially for applications involving human health, such as drug discovery and therapeutic development. Understanding the logic behind a model's predictions not only increases the confidence of developers and users in the model's inference but also makes it possible to hold AI models and their predictions accountable. For example, analytical tools like SHAP can be used to assess the influence of individual inputs on the predicted label and identify models whose prediction methods clearly contradict biological intuition, thereby filtering out inappropriately trained models that merely overfit noise in the training dataset. Another approach is to use importance metrics (e.g., attention scores) to quantify the contribution of protein substructures to the prediction, which could help determine significant local regions.

For instance, in vaccine development, identifying regions with the greatest impact on immunogenicity could help pinpoint potential antigenic epitopes^34^. In addition to this, data are crucial factors influencing the reliability of deep learning models. In the absence of a groundbreaking deep learning model, enhancing the quality and quantity of protein data is a promising path for further developing more powerful models and applying them to real‐world scenarios. Existing databases provide considerably comprehensive sequence and (experimental or predicted) structural information, while alignment with function‐relevant properties, as well as alignment across different modalities, remains limited. This calls for deep collaboration among researchers from different disciplines, such as using biostatistical methods to align proteins with genomic, transcriptomic, and proteomic datasets, which could supplement features related to protein metabolism and function from multiple perspectives and enable investigation using multimodal models. Meanwhile, protein functions also depend on external factors (e.g., pH, temperature). Although there is a scarcity of directly labeled experimental data for such conditions^20^, it is possible to align proteins with the optimal growth temperatures of their source species (e.g., bacteria) to provide approximate labels and then leverage advanced techniques such as weak supervision to assist model learning. Special attention must be paid to sensitive biological data, such as viral proteins, which could not only be used to help researchers develop targeted vaccines but also be misused for training mutation scoring models to enhance undesirable properties of the virus, such as transmissibility and immune evasion. Therefore, the risks should be evaluated before the data are made public. Additionally, similar to the editing techniques in large language models for removing toxic knowledge, unlearning operations can also be applied to pre‐trained protein models to maintain overall performance while significantly reducing predictive capability for harmful properties^150^.

In summary, while deep learning has brought protein engineering to an impressive milestone, the journey ahead offers opportunities to refine the exploration process. To progress toward these goals, future research should prioritize interdisciplinary collaboration, aiming not only to refine methodologies but also to broaden the scope of research resources. Moreover, it is crucial to acknowledge that in successful applications, biological expertise cannot and should not be replaced by models. Rich domain experience is essential, from identifying valuable template proteins to assessing engineering outcomes. Therefore, the key to transitioning from promising deep learning applications to real‐world impacts in protein engineering lies in interdisciplinary collaboration, driving the long‐term development of this field.
